# A Vestibular Challenge Combined with Calcitonin Gene-Related Peptide (CGRP) Promotes Anxiety-Like Behaviors

**DOI:** 10.1523/ENEURO.0270-23.2024

**Published:** 2024-07-24

**Authors:** Shafaqat M. Rahman, Catherine Hauser, Stefanie Faucher, Elana Fine, Anne E. Luebke

**Affiliations:** ^1^Department of Biomedical Engineering, University of Rochester, Rochester, New York 14627; ^2^Department of Neuroscience, Del Monte Institute of Neuroscience, University of Rochester Medical Center, Rochester, New York 14642

**Keywords:** anxiety, calcitonin gene-related peptide (CGRP), mouse models, rotarod and elevated plus maze, vestibular, vestibular migraine

## Abstract

Motion-induced anxiety and agoraphobia are more frequent symptoms in patients with vestibular migraine (VM) than migraine without vertigo. The neuropeptide calcitonin gene-related peptide (CGRP) is a therapeutic target for migraine and VM, but the link between motion hypersensitivity, anxiety, and CGRP is relatively unexplored, especially in preclinical mouse models. To further examine this link, we tested the effects of systemic CGRP and off-vertical axis rotation (OVAR) on elevated plus maze (EPM) and rotarod performance in male and female C57BL/6J mice. Rotarod ability was assessed using two different dowel diameters: mouse dowel (*r* = 1.5 cm) versus rat dowel (*r* = 3.5 cm). EPM results indicate that CGRP alone or OVAR alone did not increase anxiety indices. However, the combination of CGRP and OVAR did elicit anxiety-like behavior. On the rotarod, CGRP reduced performance in both sexes on a mouse dowel but had no effect on a rat dowel, whereas OVAR had a significant effect on the rat dowel. These results suggest that only the combination of CGRP with vestibular stimulation induces anxiety-like behavior and that CGRP affects the dynamic balance function in mice depending on the type of challenge presented. These findings suggest that anxiety-like behaviors can be teased out from imbalance behaviors in a mouse model of “migraine.” Future studies are aimed to determine if CGRP receptor antagonists that have been effective treating migraineurs and mouse “migraine” models may also reduce the anxiety observed in migraine.

## Significance Statement

Anxiety is very common in patients with dizziness and migraine. Elevated calcitonin gene-related peptide (CGRP) levels have been linked to migraine symptoms of increased light and touch sensitivity in mice and humans, and we wondered if a systemic injection of CGRP into mice would increase anxiety and imbalance and if mice further exposed to a vestibular stimulus would have their anxiety measures sharpened. We observed that CGRP alone increases imbalance, but not anxiety-like behaviors. However, the combination of CGRP with a vestibular challenge (VC) increased anxiety-like behaviors, whereas a VC alone was ineffective, suggesting that anti-CGRP signaling therapies may be effective for the treatment of anxiety-like behaviors.

## Introduction

Anxiety is a common burden that arises with persisting dizziness in patients with vestibular disorders (Bigelow et al., 2016; Balci and Akdal, 2020; Huang et al., 2020). Moreover, patients with vestibular migraine (VM) have been reported to exhibit severe anxiety and neuroticism toward their condition based on outcomes from the Dizziness Handicap Inventory, Beck depression and anxiety scales, and Short Form health surveys (Ak et al., 2022). Elevated levels of anxiety are also associated with a 53% increased likelihood of falls based on a meta-analysis of 18 studies linking anxiety as a risk factor for falls (Hallford et al., 2017). Fear of falling is largely detrimental to balance performance in older adults and vestibular patients.

The neuropeptide calcitonin gene-related peptide (CGRP) is involved in migraine and VM and is known to cause pain, various hypersensitivities, and anxiety (Hoskin and Fife, 2021; Russo and Hay, 2022). Recent studies have shown CGRP to induce light-aversion, tactile sensitivity, nociceptive squinting behaviors and motion sickness behaviors (Wang et al., 2021, 2022; Rahman and Luebke, 2024). While motion-induced anxiety and agoraphobia—a fear of one's surroundings—are more frequent symptoms in patients with VM than migraine without vertigo (Kutay et al., 2017), it is not known if mice with CGRP-induced motion sensitivity would exhibit higher “anxiety”-like responses and increased dynamic imbalance. Moreover, we were interested in determining if imbalance and anxiety measures are similarly affected by systemic CGRP. Thus, this paper aims to study sex-specific changes in motion-induced anxiety and balance behavior in the C57BL/6J mice that are treated with systemically delivered CGRP and a challenging vestibular stimulus. We used an off-vertical axis rotation (OVAR) as the vestibular stimulus, since OVAR can be disorienting and can promote motion sickness and nausea in rodents and human healthy controls (Furman et al., 1992; Idoux et al., 2018; Z. H. Zhang et al., 2022).

The elevated plus maze (EPM) is a behavioral test extensively used to assess anxiety-like behavior in rodent models (Pellow et al., 1985). Measurements of the time spent in open versus closed arms and the number of entries (bouts) into each arm can be used to calculate an anxiety index, an indication of anxiety-like behavior that increases with a mouse's greater presence in the closed arms (Cohen et al., 2013). The number of center crossings can serve as a measure of activity in the EPM assay as well. The EPM test is used in preclinical research to evaluate anxiety-like behaviors caused by anxiogenic drugs such as the known headache-inducing agent sodium nitroprusside (Al-Humaidhie et al., 2021), but has not yet been tested after a vestibular challenge (VC).

The rotarod assay is commonly used to assess a mouse's gait and balance on a rotating rod, and the mouse's time (latency) to fall off the rod can provide behavior insights attributed to neurological dysfunction (Deacon, 2013). Interestingly, we are the first group to assess rotarod behavior on two different dowel diameters before and after a VC.

To date, there are limited studies that have assessed CGRP-specific changes in mouse behavior that relies on vestibular cues (Y. Zhang et al., 2020) and if anxiety is exacerbated by vestibular provocation. Thus, this study's objectives are to examine the role of intraperitoneally delivered CGRP and a vestibular stimulus in anxiety and dynamic balance behavior in wild-type C57BL/6J mouse.

## Materials and Methods

### Animals

C57BL/6J mice were obtained from Jackson Laboratory (JAX 664), and mice were housed under a 12/12 h day/night cycle at the Vivarium under the care of the university's Veterinary Services personnel. They were kept in cages of up to five mice per cage and had *ad libitum* access to food, water, and bedding material. Approximately equal numbers of male (M) and female (F) mice were tested, with a total of 92 mice (47M/45F) used in these studies. All animal procedures were approved by the university's Institutional Animal Care and Use Committee and performed in accordance with NIH standards. Moreover, the experimental protocols followed the guidelines for Animal Research Reporting In Vivo Experiments (McGrath and Lilley, 2015). All studies were designed to have sufficient power to detect male/female differences, with a minimal group size of eight animals using G*Power v3.1 (Faul et al., 2007). Before beginning the experiments each day, mice were acclimated to the testing room, which was maintained at a temperature of 22–23°C, for at least 30 min. They remained in this room until the experiments were completed. Injections were given after the acclimation period. All mice tested were between 3 and 6 months of age for both the EPM and rotarod tests.

### Injections

Injections were given intraperitoneally (i.p.) using a 33 gauge insulin syringe, and behavioral testing was carried out ∼25–30 min postinjection. This delay was chosen as when peak grimace is observed after systemic CGRP injections (Rea et al., 2018). PBS was used as the diluent and control injection. Rat α-CGRP (Sigma) was injected at a dose of 0.1 mg/kg.

### EPM

EPM measures anxiety by utilizing the inherent conflict between the exploration of a novel area and avoidance of its aversive features. The mouse is placed in the middle of the maze, which is an open square (5 × 5 cm) in the center of four arms (30 cm long × 5 cm wide with two enclosed and two open). The maze consists of opaque plexiglass panels and is mounted on a stand 30 cm high. A video camera records for 5 min of the mouse exploring the maze in a dim room lighting conditions such that the open arm edge was 140 lux, the center square was 92 lux, and the closed arm was 36 lux so as not to have any light stress.

With EPM testing, prior experience with the maze can influence findings, so we staggered the test conditions (vehicle/CGRP and CGRP/vehicle) as well as tested OVAR 4 weeks after vehicle/CGRP testing. Mice were injected with either vehicle control or CGRP 20 min prior to the EPM test. Four weeks after this testing, mice were then subjected to a VC (see below, VC: OVAR) and would then be tested for EPM activity with either vehicle or CGRP injected 25–30 min prior to testing. OVAR testing was also repeated in a staggered fashion with one-half of the animals receiving vehicle and the other one-half receiving CGRP, and then 4 weeks later, the alternate substances were tested with OVAR. In this manner, we were able to conserve the novelty of the EPM testing (Walf and Frye, 2007).

**Figure 1.**
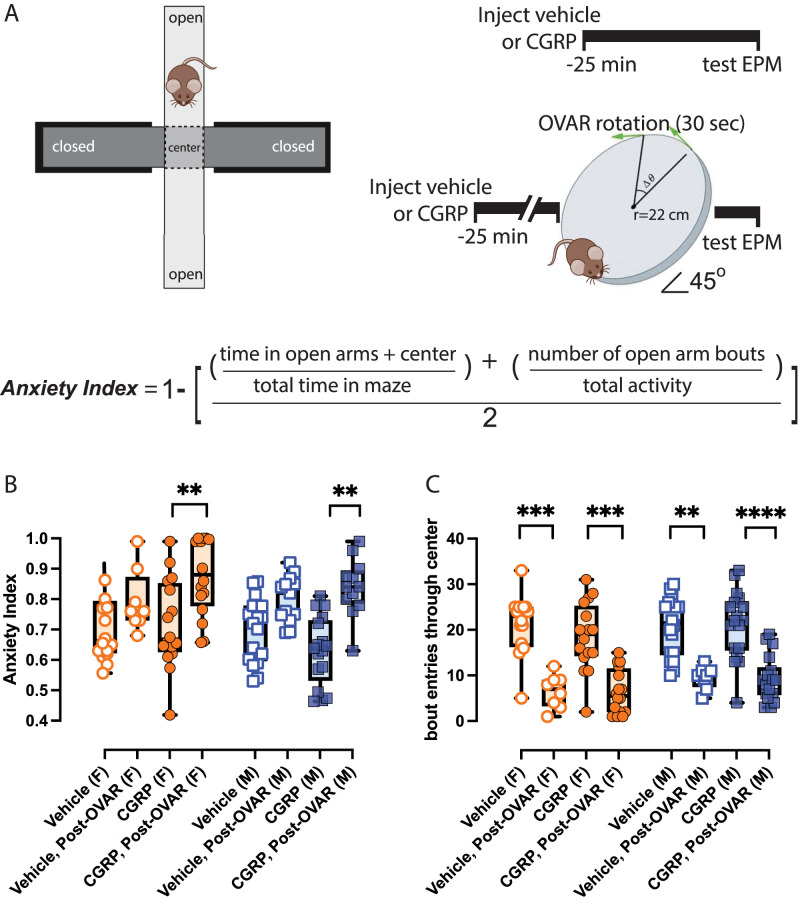
CGRP and OVAR on EPM behaviors. ***A***, The EPM and its components are shown on the left, an experimental timeline is provided on the right, and an equation for calculating the anxiety index is provided underneath. ***B***, ***C***, In 33 mice (17M/16F), two-way RM ANOVAs with Tukey’s post hoc analyses were used to evaluate ***B***, anxiety indices, and ***C***, center bouts due to either i.p. vehicle or i.p. CGRP as well as these i.p. injections to a brief (30 s) OVAR. Symbols are depicted: female (F), orange circle; male (M), blue square. ***B***, Only OVAR after CGRP injection significantly increased anxiety indices in all mice. ***C***, OVAR-induced anxiety strongly affected the number of center bouts detected in both females and males during both vehicle and CGRP testing. *P*-values and asterisks are listed: *p ≤ 0.05, **p ≤ 0.01, ***p ≤ 0.001, ****p ≤ 0.0001.

Each of the 5 min sessions was scored to determine the animal's tendency for exploring enclosed versus open arms, and the time spent in each area and the number of bouts into each arm are recorded. An anxiety index per animal is computed per experimental condition (Fig. 1*A*). Following the recordings, the mouse is returned to its home cage. The maze is cleaned between subjects using an animal disinfectant (rescue).

### Rotarod

**Figure 2.**
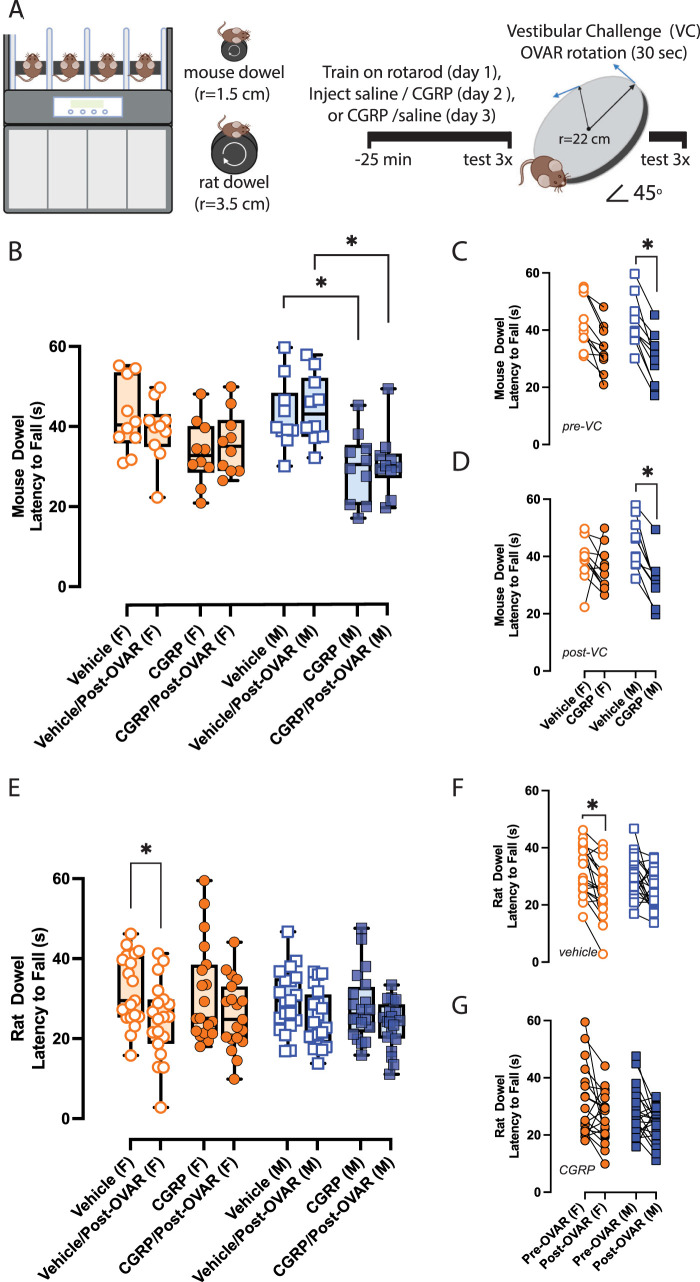
CGRP and OVAR on rotarod configured with two different dowels. ***A***, Rotarod configured with a mouse or rat dowel is shown, and an experimental timeline highlighting training and experiment days is provided. Twenty mice (10M/10F) were tested with the mouse dowel, and 39 mice (20M/19F) were tested with the rat dowel. Two-way RM ANOVAs with Tukey’s multiple-comparisons test was used to evaluate maximum LTF. ***B–D***, A disrupted pre-VC rotarod ability due to CGRP is observed in males (Fig. 2*C*). Again, after OVAR, there was a significant difference in males between i.p. vehicle and i.p. CGRP. ***E–G***, Contrary to mouse dowel studies, rat dowel studies indicated an effect of OVAR but not systemic CGRP. OVAR reduced rotarod ability in female mice treated with i.p. vehicle Again, in female mice specifically, maximum LTFs dropped from vehicle to vehicle/post-OVAR, but this was not seen in males. *P*-values and asterisks are listed: *p ≤ 0.05, **p ≤ 0.01, ***p ≤ 0.001, ****p ≤ 0.0001.

Dynamic balance was assessed with the rotarod (Columbus Instruments) configured with either a mouse dowel (*r* = 1.5 cm) or a rat dowel (*r* = 3.5 cm). Mice were tasked to maintain balance and gait on the different dowels rotating from 5 to 44 rpm at an acceleration step of 2.4 rpm every 4 s. Latency to fall (LTF) is measured when mice fall from the dowel. Three days of rotarod testing were performed. The first day was a training day where mice were tested for 6–8 trials. On the second day, mice were briefly trained and were then injected with vehicle control. Approximately 25 min after the injection, mice were tested for three trials (pre-VC) and were then stimulated with OVAR for 30 s. Mice were then immediately tested for three trials on the rotarod after the challenge (post-VC). Approximately 10–30 s pass in between subsequent trials during pre-VC and post-VC tests. On Day 3, the same mice were retested with the same methods but were instead injected with CGRP (Fig. 2*A*).

### VC: OVAR

In this study, a two-cage rotator (cage dimensions: 12.7 × 8.9 cm) was built to impose OVAR (60 rpm, 45° tilt from the vertical for 30 s) as a vestibular stimulus to mice during EPM and rotarod experiments. The mice are secured 22 cm from the rotational axis, and the device can rotate two mice at a time.

### Data analysis and statistics

EPM data were analyzed by assessing time spent in closed arms as a percentage, the number of bouts through the center platform, and a calculated anxiety index (equation found in Fig. 1*A*). For rotarod assessment, the maximum LTF of the three trials per testing condition was determined per mouse and indicated the mouse's best effort for that testing condition. A group average of the maximum LTFs and standard error (mean + SEM) was computed for comparisons. Statistical analyses were performed using GraphPad Prism 9.5. Three-way mixed-effects models were computed for EPM results, whereas three-way repeated measures analysis of variance (RM ANOVA) were used for rotarod studies. Tukey’s multiple-comparisons test was the preferred post hoc approach for all multivariate analyses. Unpaired *t* tests were only used when comparing differences in rotarod ability due to dowel lengths after i.p. vehicle treatment. Statistical significance was set at *p* ≤ 0.05 for all analyses. *F*-values and *p*-statistics for multivariate analyses across behavioral outcomes can be found in Table 1.

**Table 1.** Anxiety index and bouts through the center were computed using three-way mixed-effects models, while rotarod data using the mouse dowel (*r* = 1.5 cm) and rat dowel (*r* = 3.5 cm) was computed using three-way ANOVAs

Behavioral outcome	Factors	*F* (DF*_n_*, DF*_d_*)	*p*-value
Anxiety index	Vehicle versus CGRP	*F*_(1.00, 31.00)_ = 1.55	n.s.
	Pre- versus post-OVAR	*F*_(1.00, 31.00)_ = 58.50	*p* < 0.0001
	Male versus female	*F*_(1.00, 14.00)_ = 0.80	n.s.
Bouts through center	Vehicle versus CGRP	*F*_(1.00, 31.00)_ = 0.47	n.s.
	Pre- versus post-OVAR	*F*_(1.00, 31.00)_ = 188.00	*p* < 0.0001
	Male versus female	*F*_(1.00, 14.00)_ = 3.74	n.s.
Rotarod—mouse dowel	Vehicle versus CGRP	*F*_(1.00, 18.00)_ = 29.07	*p* < 0.0001
	Pre- versus post-OVAR	*F*_(1.00, 18.00)_ = 0.07	n.s.
	Male versus female	*F*_(1.00, 18.00)_ = 0.17	n.s.
Rotarod—rat dowel	Vehicle versus CGRP	*F*_(1.00, 37.00)_ = 0.17	n.s.
	Pre- versus post-OVAR	*F*_(1.00, 37.00)_ = 21.03	*p* < 0.0001
	Male versus female	*F*_(1.00, 37.00)_ = 1.88	n.s.

For all multivariate analyses, we examined the following three factors: treatment (i.p. vehicle vs i.p. CGRP), VC (pre- vs post-OVAR), and biological sex (male vs female). *F*-values are listed with respect to degrees of freedom (DF*_n_*, DF*_d_*) and *p*-values are listed accordingly.

## Results

### CGRP and OVAR's impact on EPM activity

In 33 (17M/16F) C57BL/6J mice, we evaluated differences in anxiety indices (Fig. 1*B*) by computing a three-way mixed-effects model examining the effects of biological sex (male vs female), VC (pre-OVAR vs post-OVAR), and treatment (i.p. vehicle vs i.p. CGRP). The individual effects of OVAR were significant on anxiety indices [*F*_(1,31)_ = 58.5; *p* < 0.0001], but CGRP's effects and biological sex were not significant (Table 1). However, the interaction of VC × treatment was significant [*F*_(1,14)_ = 4.91; *p* = 0.04], thus warranting further investigation using Tukey's multiple-comparisons test. Tukey's test indicated that OVAR caused significant increases in anxiety indices in CGRP-treated females (adj. *p* = 0.003) and CGRP-treated males (adj. *p* = 0.008). However, OVAR did not induce significant anxiety in mice after vehicle. The anxiety indices closely correlated with time spent in the closed arms. Before OVAR, CGRP-treated females and males spent 70.7 ± 3.6% and 57.0 ± 4.5% of their time in the closed arms, respectively, and after OVAR, females and males spent 92.9 ± 1.5% and 85.7 ± 2.2% of their time in the closed arms. Additionally, we quantified the number of bouts through the center platform as an indicator of movement, because each bout indicates one instance where a mouse moved from an open to a closed arm and vice versa. We computed a three-way mixed-effects model on center bouts, and also examined an effect of OVAR (*F*_(1,31)_ = 188, *p* < 0.001), but not CGRP, biological sex, or the interactions of these factors. Prior to OVAR, CGRP-treated females exhibited 18.6 ± 1.9 center bouts that decreased to 6.4 ± 1.2 after the OVAR stimulus (adj. *p* = 0.0002). Likewise, CGRP-treated males exhibited 22.2 ± 1.5 center bouts that decreased to 7.3 ± 1.0 center bouts after OVAR (adj. *p* < 0.0001). We similarly saw this reduced activity in these mice tested after i.p. vehicle, highlighting an OVAR effect after vehicle testing that was not seen in anxiety indices. Before OVAR, vehicle-treated females exhibited 21.1 ± 1.6 center bouts that decreased to 6.5 ± 1.3 center bouts after OVAR (adj. *p* = 0.0003), and vehicle-treated males exhibited 20.5 ± 1.6 center bouts that decreased to 11.2 ± 1.2 center bouts after OVAR (adj. *p* = 0.005). Generally, OVAR's effects led to a 3.2× and 1.8× reduction in center bouts for vehicle-treated females and males, respectively and a 2.9× and 3.0× reduction in CGRP-treated females and males. These findings indicate that mice sensitized to systemic CGRP's effects can experience significant anxiety-like behavior when subjected to a VC such as OVAR. Notably, while center bout activity is diminished after OVAR in mice given vehicle and CGRP injections, this anxiety-like response is not significantly pronounced with vehicle injections and only significant after CGRP injections.

### Differences in mice rotarod ability tested on different dowel diameters

While OVAR is used as the primary VC, we wanted to observe how balance function differs when mice perform rotarod on different dowel diameters (1.5 vs 3.5 cm radius). We hypothesized that mice would be further challenged on the rat dowel, because to maintain balance on a larger diameter dowel rotating at the same parameters (in revolutions per minute) as a smaller dowel, a greater distance of the larger dowel's circumference must be displaced per second. We calculated that the mouse had to travel ∼2.3× more distance on the rat dowel per second of rotation at the lowest (5 rpm) and the highest (44 rpm) speeds, ultimately becoming more of a challenge. This difficulty was reflected in the data. We observed that i.p. vehicle-treated mice on the mouse dowel exhibited higher maximum LTFs (*n* = 20, 42.62 ± 1.93 s) compared with mice tested on the rat dowel (*n* = 39, 30.75 ± 1.33 s), highlighting a difference in best performance by −11.87 ± 2.31 s when naive mice are challenged to do rotarod on a larger dowel diameter (unpaired *t* test, *t* = 5.13; df = 57; *p* < 0.0001). Due to this disparity in best performance between dowel lengths, we attempted to calibrate the rat dowel so that instead of matching the mouse dowel's revolutions per minute, it would match the displaced distance per second. We dedicated 20 naive, age-matched mice (10M/10F) to this task and observed mice to exhibit significantly higher maximum LTFs (greater than 90 s on average per trial) when rat dowel parameters were altered (data not shown). We surmised that mouse gait/imbalance cannot be easily tuned by modulating the displaced distance of the dowel and that other factors must be at play (e.g., attention and position of paws in respect to curved dowel surface) and thus did not explore this calibration further.

### CGRP and OVAR's effects on mouse dowel rotarod

Twenty mice (10M/10F) were assessed for rotarod ability on the mouse dowel (*r* = 1.5 cm) after i.p. vehicle or i.p. CGRP injections, before and after OVAR as the VC. We computed a three-way ANOVA on LTFs—the time it takes for mice to fall off the dowel—and examined the effects of biological sex, treatment, and a VC, as similarly done for anxiety indices. CGRP's effects were significant on mouse dowel LTFs [*F*_(1,18)_ = 29.1; *p* < 0.0001; Table 1]. OVAR, biological sex, and the interaction of these factors were not significant.

In males prior to OVAR, CGRP significantly reduced their rotarod activity by 13.0 ± 3.3 s compared with their vehicle tests (adj. *p* = 0.04). Post-OVAR outcomes in males also indicated a 13.6 ± 3.2 drop in rotarod activity from vehicle to CGRP tests (adj. *p* = 0.03). In females prior to OVAR, we only observed a trend in which CGRP decreased rotarod activity by 9.02 ± 2.5 s compared with their vehicle tests (adj. *p* = 0.06). However, post-OVAR outcomes in females did not suggest a CGRP effect, as shown in Figure 2*B–D*.

### CGRP and OVAR's effects on rat dowel rotarod

Thirty-nine mice (20M/19F) were used to evaluate the effects of biological sex, CGRP, and OVAR on rotarod configured with a rat dowel (*r* = 3.5 cm). A three-way ANOVA on LTF values suggest OVAR effects were significant on the rat dowel setup [*F*_(1.0, 37.0)_ = 21.0; *p* < 0.0001], but not CGRP, biological sex, or their interactions. Tukey’s multiple-comparisons test only indicated a significant effect of OVAR in reducing LTF values by 7.5 ± 1.9 s in females after i.p. vehicle (adj. *p* = 0.01). However, no other significant comparisons were observed, as shown in Figure 2*E–G*.

## Discussion

Preclinical research in migraine—via rodent models—has provided evidence that biological sex can drive the occurrence of certain mouse surrogate behaviors that resemble clinically reported symptoms, known to arise more often in women than in men. In this study, we evaluated the effects of CGRP, OVAR, and biological sex on four behavioral outcomes in the C57BL/6J mice: anxiety indices, center bouts through the EPM, and rotarod using two different dowel sizes. To reduce bias resulting from repeated behavioral testing, we staggered treatments (vehicle then CGRP or CGRP then vehicle) for all behavioral tests. Our results suggest that both female and male mice sensitized to i.p. delivered CGRP will experience significant anxiety-like behavior when exposed to a VC such as OVAR, yet do not show increased anxiety to either CGRP or OVAR alone. Consistent with our results, a prior study indicated that central, intracerebroventricular administration of CGRP from 250 to 1 µg decreases the open-field activity of adult male Wister rats in a dose-dependent manner (Kovács et al., 1999). A previous study on mutant mice, with an existing severe vestibular deficit, showed increased anxiety when tested on the EPM and was impaired on the mouse dowel rotarod. Interestingly, we did not find that anxiety indices correlated with rotarod performance in our CGRP-treated mice. With mouse dowel rotarod testing, male mice were more affected by CGRP, with no significant effect with OVAR, whereas with rat dowel testing, female mice were more affected by OVAR. In the rat dowel, CGRP treatment was not significantly different than vehicle.

We also observed reduced movement in the EPM when we measured a mouse's bouts through the center, as mice treated with OVAR were not as active as their pre-OVAR state after i.p. vehicle or i.p. CGRP injections. However, while mice were less active after OVAR, they only exhibited increased anxiety after CGRP infusion with OVAR. This motion-induced anxiety and reduced activity observed during EPM is of clinical importance, as a clear, positive correlation between anxiety, depression, and vertigo is observed in patients with vestibular disorders (Omara et al., 2022), and so studying this link in mouse models provides insight into this correlation. The EPM is also a suitable assay as a surrogate behavior for agoraphobia in mouse models for migraine, as mice spend more time in the closed arms when they exhibit anxiety-like behaviors, and mirrors patients’ tendencies to avoid open spaces during VM attacks (Kutay et al., 2017).

We did not observe an effect of biological sex in the EPM results. Interestingly, when high-anxiety rats were compared with low-anxiety rats, increased anxiety behaviors were accompanied by elevated levels of CGRP (Carboni et al., 2022). In another study, after C57BL/6J mice were exposed to an unpredictable sound stress, mice showed elevated CGRP levels in their plasma, and the CGRP receptor antagonist olcegepant abolished these stress behaviors (Viero et al., 2022).

The use of OVAR as a vestibular provocation is rarely used in rodent studies, and here we show that even a brief, vestibular stimulus induces significant anxiety in mice sensitized to CGRP and impacts balance when using a rotarod mouse dowel with 1.5 cm radius.

Anxiety is a risk factor for the progression of episodic to chronic migraine, and elevated CGRP levels have been shown to alter neuronal activity that is linked to anxiety behaviors (Durham, 2016). The results here further elucidate CGRP's role in anxiety-like indices and balance function in mouse models and should be further explored in the presence of CGRP-targeted therapeutics. Peripherally administered CGRP-signaling antagonists have been widely observed to reduce CGRP-induced migraine-like sensitivities in rodents and humans and therefore would be hypothesized to attenuate anxiety caused by CGRP in combination with OVAR (Araya et al., 2019; Raffaelli et al., 2019).

To conclude, our findings on rotarod and anxiety-like behaviors on EPM suggest that in the mouse anxiety and imbalance can be separated and support the integrated model of anxiety and postural control (Dieterich and Staab, 2017). Moreover, our findings support the claims that CGRP has a pertinent role in anxiety-like and imbalance behaviors and warrants the potential use of anti-CGRP therapies for the treatment of these symptoms.

## Synthesis

Reviewing Editor: Christine Portfors, Washington State University Decisions are customarily a result of the Reviewing Editor and the peer reviewers coming together and discussing their recommendations until a consensus is reached. When revisions are invited, a fact-based synthesis statement explaining their decision and outlining what is needed to prepare a revision will be listed below. The following reviewer(s) agreed to reveal their identity: Jinxing Wei.

Thank you for your thorough revisions to this manuscript. There are only minor concerns remaining.

1), Regarding the concentration of the calcitonin gene-related peptide (CGRP) and its duration of effect in mice, the concentration used in this manuscript is 0.1 mg/kg. What is the rationale for initiating the experiment 25 minutes or 20 minutes after intraperitoneal injection?

2), CGRP group Vs vehicle group, is there a difference in the distance traveled?

3), Concerning on figures, Figure 1A on the right side is not sufficiently neat and clear. Additionally, the notation for non-significant statistical values should be standardized; for example, 'ns' is marked in Figure 2E, whereas it is not indicated in 2F and G.
